# Selenium in Cattle: A Review

**DOI:** 10.3390/molecules21040545

**Published:** 2016-04-23

**Authors:** Youcef Mehdi, Isabelle Dufrasne

**Affiliations:** Department of Animal Production, Nutrition Unit, Faculty of Veterinary Medicine, University of Liège, 20 Boulevard de Colonster (B43), Sart Tilman 4000, Liège, Belgium; ymehdi@doct.ulg.ac.be

**Keywords:** selenium, cattle, growth, meat, milk, reproduction

## Abstract

This review article examines the role of selenium (Se) and the effects of Se supplementation especially in the bovine species. Selenium is an important trace element in cattle. Some of its roles include the participation in the antioxidant defense the cattle farms. The nutritional requirements of Se in cattle are estimated at 100 μg/kg DM (dry matter) for beef cattle and at 300 μg/kg DM for dairy cows. The rations high in fermentable carbohydrates, nitrates, sulfates, calcium or hydrogen cyanide negatively influence the organism’s use of the selenium contained in the diet. The Se supplementation may reduce the incidence of metritis and ovarian cysts during the postpartum period. The increase in fertility when adding Se is attributed to the reduction of the embryonic death during the first month of gestation. A use of organic Se in feed would provide a better transfer of Se in calves relative to mineral Se supplementation. The addition of Se yeasts in the foodstuffs of cows significantly increases the Se content and the percentage of polyunsaturated fatty acids (PUFA) in milk compared to the addition of sodium selenite. The enzyme 5-iodothyronine deiodinase is a seleno-dependent selenoprotein. It is one of the last proteins to be affected in the event of Se deficiency. This delay in response could explain the fact that several studies did not show the effect of Se supplementation on growth and weight gain of calves. Enrichment of Se in the diet did not significantly affect the slaughter weight and carcass yield of bulls. The impact and results of Se supplementation in cattle depend on physiological stage, Se status of animals, type and content of Se and types of Se administration. Further studies in Se supplementation should investigate the speciation of Se in food and yeasts, as well as understanding their metabolism and absorption. This constitute a path to exploit in order to explain certain different effects of Se.

## 1. Introduction

Selenium (Se) is a trace element that plays an important role in the health and performance of animals. In cattle, selenium deficiency can have economically significant impacts such as reduced fertility, placental retentions, and the incidence of mastitis and metritis [1,2,3,4]. The increase in fertility when adding selenium is attributed to the reduction of the embryonic death during the first month of gestation. In the immune system, selenium plays a role in the formation and the activity of helper T, cytotoxic T and Natural killer (NK) cells [5]. A selenium deficiency leads to disorders in the perinatal period altering milk quality in cows [6,7]. Selenium is a trace element that plays a role in the defence against the accumulation of hydroperoxides from cellular metabolism [8]. This biological function is accomplished through the selenoproteins, such as the glutathione peroxidase family (GPx) the iodothyronine deiodinases and the thioredoxin reductases, in which selenium is a structural component [9] These proteins have selenocysteine aminoacids (Secys) in critical positions of the active centre of the enzyme. The implications of selenium in the organism are multiple [10]. This review article examines the role of selenium and the effects of selenium supplementation, especially in the bovine species.

## 2. Selenium Nutritional Requirements in Cattle and Interaction with Vitamin E and Trace Elements

The daily nutritional requirements of selenium in cattle are estimated at 100 μg/kg DM (dry matter) for the beef cattle and at 300 μg/kg DM for dairy cows. In calves, the selenium requirements are 100 μg/kg DM per day [11,12]. For hyper muscular cattle races, an intake of 100 μg/kg DM of selenium per day seems not to cover all the requirements. In the case of Belgian Blue bulls, Guyot and Rollin [13] advocate an intake of 300 μg/kg DM per day.

Vitamin E is one of the factors influencing the dietary selenium consumption [14]. It should be noted that selenium and vitamin E antioxidant functions are interdependent. A diet low in vitamin E may increase the amounts of selenium necessary for the prevention of certain anomalies [14]. NRC [15] recommends 15 to 60 international units (IU) as the daily nutritional need of vitamin E in adult cattle. Meanwhile the daily needs for nursing calves are 40–60 IU. Nevertheless, vitamin E and selenium are involved in similar functions. Therefore, a selenium deficiency could be partially compensated by an adequate intake of vitamin E and *vice versa*. There is a close link between selenium and vitamin E status and antioxidant status [16]. Selenium and vitamin E deficiency may result in a malfunction of the thyroid metabolism, which can cause a decrease in the growth rate, reduced fertility, altered phagocytic response, and a drop in disease resistance [17].

According to Kessler *et al.* [18], diets rich in carbohydrates, nitrates, sulphates, calcium or hydrogen cyanide (clover, flax seeds) negatively influence the organism use of selenium in cattle. Sulfur (S) could decreases the absorption of selenium by steric competitiveness at a concentration over 2.4 g/kg DM. Similarly, Fe^3+^ decrease the rate of absorption of selenium. Fe^3+^ precipitates selenium to a complex form unassimilable by the enterocytes [1]. A calcium level of 0.8% DM in the feed allows an optimal apparent absorption of selenium in dairy cows in late pregnancy [19]. Garcia-Vaquero *et al.* [20], showed that calcium supplementation in cattle, with concentrations typically used in intensive production, causes a significant decrease in the selenium content in muscle. In addition, serum levels of selenium and its content in all tissues decreased in the case of high concentration of lead in the diet of the calf [21]. Iodine deficiency could exacerbate a selenium deficiency. In thyroids of cattle, iodine deficiency leads to marked induction of the selenoprotein-D1 which is accompanied by elevated GPx activity [22]. The raw materials rich in crude protein and cellulose have a positive impact on the selenium use by the assimilations rates [18].

## 3. Variations of Selenium Content in Rations, Toxicity and Deficiencies

Selenium deficiency is more of a problem geographically than is selenium toxicity [23]. The selenium content of forages varies with the type of feed, the type of soil and the region. Forages containing sulfur amino acids store more selenium than fodder. Sulfur (S) present in the methionine and cysteine is substituted by selenium to form selenomthionine (Semet) and selenocysteine. These two organic forms of selenium are the most common ones in forages. They are well absorbed by the body and accumulate in animal tissues [24]. Stems, leaves and seeds of plants are rich in selenium than the roots. In soils, selenium content varies with the soil type, texture, organic matter content and precipitation. The assimilation of the selenium by the plant is influenced by the physico-chemical factors of the soil, such as the redox status, pH and microbiological activity [10]. The selenium content in the forage also depends on the type of selenium available in the soil. For example, Selenate is absorbed ten-fold better by plants than selenite [25]. The type of soil, chemical factors and returning to a more extensive farming type and organic farming can favour the selenium deficiency appearance in cattle [26].

Adequate selenium intake can be achieved through supplementation. Some of selenium supplementation include the addition of selenium in drinking water, supplementation with mineral salts and selenium enriched yeast, injections, implants, and the use of bolus fodder grown with fertilizers enriched in selenium [18]. We must also consider the toxicity of selenium in order to not endanger the life of the animal or the consumer [27]. Its content in the diet should be based on the efficacy range to avoid accidents due to selenium deficiency or excess. According to Neve and Favier, [28] and Claude [26] the signs of selenium toxicity start to appear in the 5 to 8 mg/kg DM range. The most common forms of selenosis are chronic selenosis, referred to as alkali disease, and acute selenosis, popularly known as blind staggers [29]. In turn, marked deficiencies are observed when a content of selenium is less than 0.05 mg/kg DM in a diet [26]. The maximum selenium content standard in foods is set at 300 μg/kg DM in America and 500 μg/kg DM in Europe [30].

## 4. The Effects of Selenium Supplementation

### 4.1. The Effects of Selenium Supplementation on the Milk and Colostrum

The selenium content in the milk is an easy way to assess the selenium status of a herd. Sampling is usually done at the milk tank on the farm, allowing one to estimate an overall selenium status of the herd. Wichtel *et al.* [31] provide reference levels for the selenium status estimation from milk. These thresholds can be formulated as follows: a selenium content of less than 0.12 μmol/L defines a deficiency status, the selenium content greater than 0.28 μmol/L is adequate, and between 0.12 and 0.28 μmol/L is marginal.

According to Muñiz-Naveiro *et al.* [32], the highest selenium levels were found in milk whey (56.6%), while the lowest level was found for the fat phase (10.1%). A fraction of 55%–75% of selenium found in milk is incorporated in casein, a fraction of 17%–38% is contained in the whey and 7% are localised in fat [33]. These findings do not vary with the type of ingested selenium [34]. The selenium content in milk varies mainly depending on the selenium amounts in the diet of cows. Ivanis and Weiss, [35] derived the equation highlighting a positive correlation existing between plasma and milk selenium content: *y* = 0.37*x* − 4.2, with *x* = [Se] in the plasma (μg/L) and *y* = [Se] in the milk (μg/L). Generally, the amount of selenium in the plasma is from 3 to 5 fold higher than that found in milk. Cows supplemented with selenium by the oral route before parturition, produce colostrum 2-fold richer in selenium than non-supplemented cows (170 compared to 87 μg/L, [36]). This fact was proven by Pragon’s [37] work which also found that colostrum is 2- to 3-fold richer in selenium than milk. 

The selenium amount in milk is influenced by its content in foods depending on a season and a farming region [38]. In a study conducted during the grazing season, Ceballos-Marquez *et al.* [39] reported a marginal selenium levels in the milk of 14% of the dairy herd . This observation was related mainly to an inadequate intakes of selenium due to poor forages. Studies conducted in Belgium [40], South Korea [41], Greece [42] and Australia [38] reported selenium contents of 30, 60, 15 and 22 μg/kg in the milk, respectively. In addition, selenium present in milk helps to meet a daily portion of the selenium nutritional requirements in humans. For example, in Belgium 4% of the selenium consumed by the population comes from milk and its derivatives [40]. In South Korea, this rate raises to 7% [41].

The amount of selenium in the milk is directly proportional to the organic selenium content in the feedstuffs of cows [43,44]. Ceballos *et al.* [43], reported that on average, cows fed organic selenium have 0.37 μmol/L more milk selenium than cows supplemented with inorganic forms. The addition of selenium yeast or sodium selenite in the cow feeds increases significantly selenium content [6], the percentage of the polyunsaturated fatty acids (PUFA) and linoleic acid content in milk compared to the control [7]. The increase of these parameters is more pronounced in case of selenized yeast as compared to sodium selenite supplementation. In addition, organic selenium supplementation of dairy cows rations in a form of selenized yeasts was shown to induce selenium concentration in milk reaching levels up to 190% higher as compared to milk from cows supplemented with inorganic selenium [45]. The same difference was observed by Salman *et al.* [46] in milk and colostrum. In addition, Salman *et al.* [46] reported that lymphocyte subpopulations and phagocytosis activity of neutrophilic granulocytes were affected neither by the selenium intake nor by the different dietary supplements (sodium selenite and selenium yeast). Conversely, selenium supplementation of feedstuffs appears to have no significant effect on milk production in cows and its chemical composition (in terms of fat, protein, lactose) [6,7,44,46]. An effect on milk production has been reported when supplementation is made with selenium associated with iodine and cobalt in the form of a ruminal bolus [47], or when selenium is associated with vitamin E [4]. Eulogio *et al.* [4] reported also an increase on percentage of crude protein, solids non-fat and lactose when selenium supplementation is associated with vitamin E. This increase in milk production of the cows was not observed when the trace element association is made as an injection. Indeed, Machado *et al.* [48] did not find an effect of the injection of selenium (25 mg), zinc (300 mg), manganese (50 mg), and copper (75 mg) on milk production. Dairy cows which received an oral supplementation of selenium of 300 μg/kg DM coupled to injection of 50 mg of selenium and 300 IU of vitamin E, 21 days before calving, maintained an adequate concentrations of selenium in plasma [49] ensuring a good selenium level in milk.

### 4.2. Effects of Selenium Supplementation in Newborn Calves 

In ruminants, the transfer of selenium from cow to the newborn is done through the placenta and milk. Transfer performed via the placenta is more efficient than that made through the milk [50]. The reason may be the higher concentration of selenium in blood serum than in milk. The last months of gestation cows represent the critical period for the selenium availability. The transfer of selenium to the fetus and subsequently to the calf occurs even when cows are deficient in selenium. Cows sacrifice selenium available to them to ensure adequate intake for newborns [2]. This situation is explained by the fact that the renal selenium levels in the fetus remain unchanged whereas that of cows, at the end of pregnancy, decline [51].

The mineral selenium is not well transferred into the milk [52], and thus is not efficient in maintaining adequate selenium status in calves. Compared to mineral selenium, use of organic selenium in foods was shown to cause better selenium transfer rates in calves [43,52].

Contrary to sheep [53], selenium supplementation in calves generally has no influence on calf growth performance [36,54]. Positive effects were reported when selenium supplementation is performed in deficient calves [55]. Indeed, the authors report a significant increase in the average daily gain for calves treated by selenium injection (0.05 mg/kg at day 2, 70, 114 and 149 days) as compared to controls. It emerges from the study of Gunter *et al.* [54] that the type of selenium, namely inorganic or organic, does not have a significant impact on the birth weight, gain and mortality rates in calves. Similarly, during a supplementation at 0.08 mg/day in newborn calves, Salles *et al.* [56] reported no effect on the weight gain and the height or length development in calves. However, significant effects of selenium supplementation on the immune system of calves were recorded in the study of Salles *et al.* [56]. These authors report that high concentrations of selenium in serum induced an increase of the phagocytic activity of macrophages in 30 days old calves. An increase of the immunoglobulin concentration in calves has been demonstrated to be caused by a mineral selenium supplementation of cows before calving [36,57]. Indeed, strengthening of their immature immune system helps them to resist to the inadequate environmental conditions. At temperatures below 14.6 °C, newborn calves present sensitivity to cold [58]. To cope with this stress, the thermogenic response is generated by the metabolism of brown adipose tissue. The metabolism of the adipose tissue is regulated by T3 hormone which is a seleno-dependent hormone [36]. In cases of severe selenium deficiency in calves, it is advisable to couple a selenium injection together with self-service access to a dietary selenium source. Such practice helps to rapidly restore an adequate selenium status and to fix it for a few months [59]. In addition, subcutaneous injection of calves deficient in selenium linked to the selenium supplementation diet improves the activity of GPx [60].

It can be concluded that selenium does not play a direct role in promoting growth in calves. However, it helps to remove all constraints that may delay or inhibit growth. Selenium injection is considered a useful corrective tool to quickly establish an appropriate status of selenium of calves. However, it should not be considered as a permanent source of selenium intake.

### 4.3. Selenium Content in the Blood

In blood, the selenium is bound to the α and β-globulins, LDL (low density lipoprotein) and VLDL (very low density lipoprotein) and albumin [61]. Ten selenium atoms as selenocysteine are included in the selenoprotein-P which is a selenoprotein supplier of selenium to body tissues [62]. According to Dargatz *et al.* [63], the rate of adequate selenium in the blood of cattle is between 0.08 and 0.16 mg/L. In plasma, the selenium is mainly found in the albumin fraction. According to Villard *et al.* [64], the appropriate level in plasma selenium is between 51 and 85 μg/L. To achieve this adequate rate, additional dietary intake of 0.5 mg/kg DM may be enough [14]. An increase in the concentration of selenium in the diet is followed by an increase of the selenium concentration in the serum within two to six days after supplementation [65,66].

Selenium supplementation in the diet of calves [67], heifers [60] or fattening bulls [68] is usually manifested by an increase of the selenium content and GPx activity in the blood. This increase is more pronounced upon the addition of selenium in organic form in the food compared to inorganic form [7,60]. The difference in effect between these two forms of selenium is estimated at 20% for the blood selenium content and 16% in the GPx activity [69]. According to Hall *et al.* [70], cows fed a supranutritional selenium-yeast supplement during the last 8-weeks of gestation had higher selenium concentrations in whole blood (overall 52% higher) and serum (overall 36% higher) at 48 h and 14 day of lactation. Organic selenium supplementation on dairy cows induce lower serum cholesterol concentrations and higher α-tocopherol/cholesterol ratios at calving and at 48 h compared with control [70].

The selenium increases are produced gradually during a supplementation of the diet (2 mg of organic or inorganic selenium) [45]. A combination of injections and diets supplemented with selenium help maintain normal blood selenium content and GPx activity in heifers during the first weeks in fattening units [60].

### 4.4. Effects of Selenium on Growth Performance

Selenium is involved in the metabolism of thyroid hormones. A selenium-deficient diet causes a reduction of triiodothyronine (T3) and an increase of the tetraiodothyronine (T4) and a decrease in the ratio T3/T4 levels in blood [71,72]. These effects can influence growth rates since T3 is an active form of T4 which is known to be involved in the growth mechanisms. The activation of the T4 is done using the enzyme 5-iodothyronine deiodinase. This seleno-dependent selenoprotein is one of the last proteins to be affected in the event of selenium deficiency. This delay could explain the fact that several studies that have explored various ways of selenium supplementation do not show any significant effect of selenium supplementation on growth, weight gain of calves, cows or bulls. The subcutaneous injection [55] or intramuscular [73] selenium has also no effect on growth performance. Similar results are reported in a mineral selenium supplementation (sodium selenite) or organic (selenized yeast) in the cow and calf diets [54,74,75]. According to Chorfi *et al.* [60], the selenium source has no effect on the dry matter intake and weight gain in heifers. When using a diet concentrates composed of cereals produced with fertilizers enriched in selenium, Mehdi *et al.* [68] did not find a significant impact of such soil enrichment strategy on the growth of fattening bulls. Similarly, supplementation with an intra-ruminal bolus of the cow had no influence on the birth weight nor the average daily gain (ADG) of calves [73]. However, Wichtel *et al.* [76] reported an increase of 20% of ADG when using intra-ruminal bolus in calves aged 5 months. Results also different on the ADG and growth of calves having a low selenium status were recorded by Castellan *et al.* [55]. An increase in growth was recorded after addition of selenium in the diet of cattle suffering from hypocupremia [77]. In this context, we can assume that the response to selenium supplementation depends on the current selenium status (low or adequate) in animals. Growth in cattle can be influenced indirectly by selenium deficiency. This is caused by a dysfunction of muscle pathologies such as white muscle disease. As a result, it is necessary to include selenium in feedstuffs.

Selenium enrichment of the diet of young fattening bulls and steers had no significant effect on weight at slaughter and carcass yield. These effects were observed in the studies of Lawler *et al.* [78] with organic selenium (selenized yeast), Netto *et al.* [79] with the mineral selenium and Mehdi *et al.* [68] with the cereals produced from the fertilizer selenium enriched soil.

### 4.5. Effect of Selenium Supplementation on Cattle Reproduction

Selenium deficiency presents a factor favouring the appearance of perinatal metritis and retention of placenta in dairy cattle [1,3]. In addition, selenium deficiency can cause a malfunction of the testosterone and spermatozoon synthesis, which causes infertility in males [80]. Selenium is known to influence the gross and histological morphology of the testis [81]. Selenium deficiency is often characterized by reduced spermatozoon motility due to the fragility of its intermediate piece [82]. Some selenoproteins were localized in the testes as selenophosphate synthase-2 (SPS-2) and the mitochondrial capsule selenoprotein (MCSeP) [83,84]. An increase of the selenium content in the testes of cattle was reported during the supplementation with selenium enriched cereals [68], mineral selenium (selenite) [85] and organic selenium (yeast) [78].

The increase in fertility when adding selenium can be attributed to the reduction in embryonic death in the first month of gestation. Indeed, it appears from the analysis of Ceko *et al.* [86] on expression of the selenoprotein gene that GPx-1 is significantly increased in granulosa cells of large healthy follicles. These authors conclude that selenoproteins have an antioxidant role during the belated follicular development. Similar results were reported in goats [87]. Wu *et al.* [87] found that s nanoselenium supplement could promote the development of secondary follicles in cashmere goats.

Selenium supplementation may reduce the incidence of metritis and ovarian cysts during the postpartum period [88]. Spears and Weiss [1], reported that selenium supplementation of dairy cows decreased the incidence of retained placenta. Furthermore, it appears from the study of Komisrud *et al.* [89], that selenium supplementation in dairy cows deficient in selenium may improve the success rate to first service. However, according to Gunter *et al.* [54], the source of selenium (sodium selenite or selenized yeast) has no effect on the conception rate and the calving interval in cows.

The literature related to the selenium supplementation in cattle reported its contradictory effects on reproduction. These contradictions could be due to the severity of the deficit, conditions of supplementation and the system’s ability to enzymatic synthesis under these conditions [2]. It is also important to note that in cattle, chronic selenosis lowers fertility by supporting the growth of ovarian cysts and prolonging anoestrus [29].

### 4.6. Effect of Selenium Supplementation on the Cattle Health

The nutritional status of dairy cows is widely recognized as being closely linked to the maintenance of optimal immune function and health [8]. Selenium (Se), iodine (I), zinc (Zn) and copper (Cu) deficiencies in cattle have been often associated with diseases [23,90]. Machado *et al.* [48] found that administration of three subcutaneous injections of trace minerals (300 mg of zinc, 50 mg of manganese, 25 mg of selenium, and 75 mg of copper) had a positive impact on udder health, decreasing linear somatic cell count scores, the incidence of subclinical mastitis, and the incidence of clinical mastitis. According to Hall *et al.* [70] feeding selenium-replete cows during late gestation a supranutritional selenium yeast supplement improves antioxidant status and immune responses after calving. There is a relationship between selenium content in the diet and mastitis frequency in cows, knowing that the phagocytic activity of neutrophils is the primary defense mechanism against mastitis [30]. Selenium affects the innate and the adaptive immune responses of the mammary gland through humoral and cellular activities [91]. According to Finch *et al.* [92], several researchers have demonstrated a significant reduction in the incidence of mastitis in dairy cows after they were supplemented with selenium and/or vitamin E. According to Eulogio *et al.* [4] the performance and economic feasibility of the use of selenium and vitamin E allowed to obtain a profit margin of 0.21 $US per animal per day. However, Ceballos-Marquez *et al.* [39] did not reveal the presence of link between selenium status and intra mammary infections and clinical mastitis.

According to Petrie *et al.* [5] selenium dietary supplementation can improve expression of various humoral and cellular immune responses. Bulls that received selenium (yeast) and vitamin E had higher NK cell cytotoxicity than control Nellore bulls [93]. Selenium supplementation causes increased expression of natural killer (NK) cells in the spleen cells. There is an effects of selenium supplementation on passive transfer of immunity from the dam to the calf.

Hefnawy *et al.* [2] report that selenium supplementation of cows induces a high concentration of IgG in the serum and colostrum. Higher levels of IgG in the serum were also recorded in their calves. Kamada *et al.* [94] found that selenium supplementation (selenite) of colostrum increases IgG absorption by new-born calves. The redox balance of the colostrum plays an important role in the passive transfer of immunity from the colostrum to the calf. The findings of Abuelo *et al.* [95], suggest that selenium supplementation enhanced the antioxidant properties of the colostrum. Similarly, Sordillo *et al.* [3] reported a decrease of the phagocytic ability of blood and milk neutrophil to kill pathogens in dairy cows with a selenium deficiency. The opposite situation was reported for neutrophils cows having a higher status of selenium. High levels of selenium may cause both the inhibition of tumor cell proliferation and improvement of *in vivo* and *in vitro* immunity [5]. A study about glucose tolerance in dairy cows [96], show that cows supplemented parentally with selenium and vitamin E before calving showed improved insulin sensitivity during the first week of lactation.

Nevertheless, while selenium is an essential micronutrient for various immune mechanisms, according to Nair and Stanley [97] an excess of selenium can have a detrimental effect on some immunological functions. In general, the excessive supplementation with antioxidant can increase the production of reactive oxygen species (ROS) [96,98].

### 4.7. Distribution of Selenium in the Muscles and Organs

The selenium content in the forages and grass depends directly on the selenium content in the soil [99]. This creates a difference in the levels of selenium in animal meat according to breeding areas. The selenium content in the meat also varies among species because of the morphology of the digestive organs and the metabolism of selenium. The absorption of selenite is about 80% in monogastric animals and poultry, whereas this rate is only 29% in ruminants [30]. In ruminant (ewes), Galbraith *et al.* [100] showed that organic selenium as SeMet was incorporated to a greater extent into rumen micro-organism than inorganic selenium sources and resulted in less elemental selenium formation. According to Galbraith *et al.* [100] the oral bioavailability of organic SeMet should be greater compared with inorganic selenium sources because of greater rumen microorganism incorporation of selenium and decreased formation of elemental selenium by rumen micro-organism. The aspect relating to selenium metabolism and transport have been treated in detail in the review of Burk and Hill, [101] and will not be detailed in this section.

During a selenium supplementation in animals, the effect of enrichment on the selenium content in the muscles and organs is different. Part of the absorbed organic selenium is incorporated directly into the muscle proteins, while the inorganic selenium is mainly used for the synthesis of selenoproteins [102,103]. Inorganic selenium supplementation provides no significant difference in the selenium concentration in the muscles [78,79,85]. During a selenite supplementation, most of the selenium is incorporated in selenoproteins. During supplementation with organic selenium, a part follows the same metabolic pathways as selenite, but a certain amount is deposited directly and non-specifically in muscle proteins. There are differences in the absorption of selenium contained in yeast by the body. Kieliszek *et al.* [104] noted that binding of selenium by microbial cells largely depends on the culture conditions, the concentration of selenium in experimental medium, and the organisms used. The source of organic selenium leads to differences in impact of selenium supplementation. In a meta- analyse of the effect of selenium supplementation, Bermingham *et al.* [105] report that for animal species selenium-enriched foods are more effective than selenomethionine at increasing GPx activity, which could give result in selenium content in the kidneys.

The highest density of selenium content in cattle is found in the kidneys, while in muscles it is present at the highest amounts. The first organs affected by selenium deficiency are heart, skeletal muscle and liver [30]. When selenium supplementation, the kidneys and the liver have the highest selenium contents [68,78,85]. Kidneys are organs which store the highest amount of selenium followed by the liver, the testis and the lungs [78,85,106]. Supplementation with the sodium selenite causes selenium to be distributed in the whole organism. However, most of the absorbed selenium is found in the liver [107]. During the absorption of selenium, a large proportion is directed toward the liver, which is considered selenium storage organ. Selenium accumulates in the liver, in the case of an excess of selenium compared with needs, a part of the selenium stored in the liver is excreted via bile. Another most important part is excreted via the kidneys. When selenium is in excess, selenide is polymethylated and excreted (dimethyl selenide in breath and faeces; and the cathion (CH_3_)_3_Se^+^ in urine) [103,108,109]. 

### 4.8. Effect of Selenium Supplementation on the Meat Quality, Chemical Composition and Fatty Acids Profile

Lipid oxidation is the main cause of deteriorating meat quality [110]. Oxidation reactions in the meat are the most influencing factor in the quality of the meat. These reactions affect the color, flavor, texture and nutritional value of meat [111]. Joksimović-Todorović *et al.* [112] report that recent research has shown that selenium has an effect of preserving sensory characteristics of meat and its texture among domestic animals. Use of selenium-enriched cereals was found to be an interesting way of selenium supplementation of fattening bulls [68]. This kind of supplementation had no significant effect on the color, pH, water loss, tenderness and oxidative rancidity of meat. In addition, this type of supplementation induced a decrease in the fat content of meat [68]. In the study by Netto *et al.* [113] a reduction of the cholesterol content in the meat was reported during a supplementation with mineral selenium. Glutathione peroxidase 4 (GPx4) is an essential antioxidant selenoenzyme well known to protect against lipid peroxidation [114]. Further, selenium may play a role in the alteration of lipid metabolism. Cholesterol is a biologically important compound that is present in animal products. The cholesterol content in meat and meat products is variable, it is generally less than 70 mg/100 g of meat [115]. The cholesterol content in edible offal is higher compared to meat. A decrease of the content of cholesterol in meat when adding selenium would be a beneficial effect of selenium supplementation. The meat would be dietary and healthy. Indeed, the oxidation of cholesterol (COP) generates compounds that were found to be cytotoxic, mutagenic and carcinogenic. Cholesterol oxidation products are also considered to be a main trigger atherosclerosis [115]. Nevertheless, the results concerning lipid decrease [68,113] were not consistent with those reported in other studies in cattle [85,116,117], rabbit [118] or pigs [119,120] for which no difference was observed in lipid when adding selenium. Further comparative studies using different sources and selenium levels, as well as determination of various types of lipids in meat are necessary.

During selenium supplementation in cattle, the sum of saturated fatty acids, monounsaturated and polyunsaturated for meat was not influenced by the source of selenium (sodium selenite or selenized yeasts) [24]. Netto *et al.* [113] report similar results except for linoleic and palmitic acid. These authors reported an increase of these two fatty acids amounts thanks to organic selenium supplementation (2 mg/kg MS) in the diet of fattening steers. Selenium source was reported to have no effect on the fatty acid profile of the meat. However, organic selenium is known to be linked to a higher selenium content in the meat compared to the inorganic selenium [24].

The different results reported in the literature regarding the composition of the meat can be attributed, as for tenderness, to different rates of incorporation of selenium, sources of selenium, type of selenium and the administration routes.

## 5. Conclusions

Selenium plays an important role in the health and production of cattle. Selenium deficiency has direct or indirect negative effects on growth, production and health of cattle. Excessive selenium supplementation can lead to toxicity. The availability of selenium in sufficient quantities in the diet ensures the proper functioning of the immune and reproduction systems. The presence of selenium in the diet provides a high content of selenium in the milk and meat, which is essential for humans. There are several modalities of selenium supplementation in cattle. The impact and results of selenium supplementation in cattle depend on physiological stage, selenium status of animals, type and content of selenium and types of selenium administration. Yeast accumulate organic forms of selenium but also accumulate selenium as mineral. Organic forms and their quantities contained in yeast are different, depending on the environment of the yeast culture, organisms used and the concentration of selenium in the medium, which may affect the impact of selenium supplementation. Further studies on selenium supplementation should investigate the speciation of selenium in food and yeasts, as well as our understanding of their metabolism and absorption. This constitutes a track to exploit in order to explain certain sometimes contradictory effects of selenium supplementation like its concentration in muscles and organs and decreased lipid levels in the muscles.
